# Thermal Conduction Suppresses Cracks in PDMS Wrinkling by Plasma Oxidation

**DOI:** 10.1021/acs.nanolett.4c05019

**Published:** 2024-12-07

**Authors:** Zain Ahmad, Begoña Parias M. R, Helen Barr, João T. Cabral

**Affiliations:** Department of Chemical Engineering, Imperial College London, London SW7 2AZ, United Kingdom

**Keywords:** patterning, wrinkling, crack formation, PDMS, plasma

## Abstract

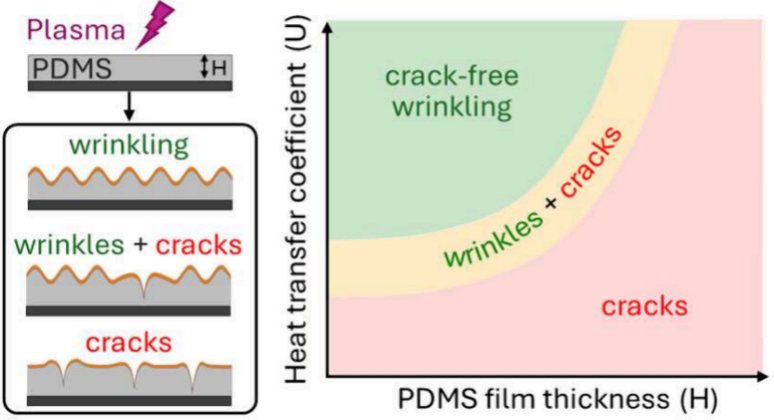

We report a facile approach to suppress intrinsic crack formation during wrinkling of plasma-oxidized polydimethylsiloxane (PDMS) films, removing a major hindrance in the practical use of these ubiquitous, functional surface patterns. A combination of high heat transfer coefficient (HTC) of the film substrate and low PDMS thickness is shown to consistently yield crack-free wrinkling of glassy skin and PDMS bilayers. Employing optical and atomic force microscopy, light scattering, thermal measurements, and heat transport and stress calculations, we demonstrate that our findings hold for a range of glass, plastic, metal, and layered support materials and plasma processing conditions. Subsuming the PDMS contribution to thermal conduction, an overall HTC threshold of ∼800 W/(m^2^ K), for typical plasma exposures, for crack-free wrinkling is obtained. While suppressing cracks, this simple approach retains the functionality of the glassy skin of plasma-oxidized PDMS.

Surface instabilities in soft materials lead to the formation of complex morphologies, from nano to macro scales, providing insight into the emergence of shape and function in Nature.^1−5^ Highly regular wrinkling patterns emerge when a stiff thin film, bonded to a thick softer material, is subjected to a compressive mechanical stress.^6−9^ Periodic surface undulations with prescribed wavelengths λ and amplitudes *A*, and in-plane orientation, from uniaxial to isotropic, as well as higher order modes and complex morphologies have been demonstrated, unlocking technological applications in controlled wetting and spreading,^10^ optics,^11^ flexible electronics,^12^ sensors,^13^ thin film metrology,^14^ drag reduction,^15^ antibacterial action,^16^ and biofouling mitigation.^17^ Polydimethylsiloxane (PDMS) elastomers are commonly employed in wrinkling due to a range of physical and chemical properties, including optical transparency, near incompressibility and high elongation at break, thermal and chemical stability, and hydrophobicity.^18,19^ PDMS wrinkling approaches generally involve (i) the formation of a stiff-soft bilayer (by film lamination, deposition, metal evaporation, plasma exposure or UV ozonolysis, polymerization, surface modification, etc.) and (ii) the application of external fields (mechanical, shrinkage, evaporation and sorption, thermal, light, etc.).^1−3,20,21^ PDMS plasma and UVO oxidation creates a stiff, glass-like surface skin, by substituting hydrogen and methyl groups with hydroxyl groups and oxide links, with a range of intermediates, resulting in the progressive, directional densification of an SiOx layer with thickness *h* ∼10 nm (plasma) to ∼μm (UVO),^22−26^ providing a facile and scalable route to such bilayer structures.

A major challenge associated with PDMS wrinkling remained the stubborn appearance of surface cracks, restricting the usable patterned areas, hindering reproducibility, and degrading function.^27,28^ Cracks are surface tears that appear on the glassy skin layer generally along the direction of applied stress (transversally to surface undulations) due to its relative inelasticity, providing a competing stress relaxation mechanism that disrupts patterning. A number of strategies sought to suppress crack-formation in surface-oxidized PDMS, generally by modifying the nature of the stiff skin,^29−32^ plasma processing,^33,34^ or the method of application of mechanical strain.^6,33,35,36^ The latter mitigates “extrinsic” cracks, defined as those caused by compression mechanics, but not “intrinsic” cracks, which appear during plasma exposure and glassy skin formation, in the absence of external mechanical stress.

Here, we examine the role of the thermal excursion experienced by PDMS films during and after plasma exposure on bilayer stress and the subsequent formation of “intrinsic” cracks. We hypothesize that the interplay of thermal expansion and glassy skin formation can provide opportunities to modulate and suppress crack formation.

PDMS films of thickness 10 ≤ *H* ≤ 1000 μm were fabricated by spin coating onto silicon (Si) substrates and subsequently exposed to O_2_ plasma, as depicted in Figure 1a. Selected films were transferred to distinct substrates and exposed to plasma (SI Figure S1). Isotropic surface wrinkling emerges following plasma exposure,^37,38^ with wavelength λ and amplitude *A* governed^39−41^ by plasma process parameters: λ = 2π*h*(*E̅*_f_/(3*E̅*_s_))^1/3^ ≈ 1 μm where *E̅*_f_ ≈ 10 GPa and *E̅*_s_ ≈ 1.5 MPa are the plane strain moduli of the glassy skin and PDMS, respectively (where *E̅* ≡ *E*/(1 – ν^2^), *E* is Young’s modulus and ν is the Poisson ratio, respectively ∼0.2 and 0.5). Extensive *intrinsic* cracking generally takes place alongside, at ∼mm length scales (Figure 1b), and near cracks, the wrinkling pattern isotropy is generally broken,^2,42^ reflecting a change in the local stress field. Our first observation (Figure 1c) is that the plasma exposure of a single PDMS film placed across PDMS and copper substrates yields extensive cracking on the thermal insulator and crack-free wrinkling on the thermally conducting support. Further, a gap imposed by a PMMA microparticle (∼130 μm) between a PDMS film and its silicon support, another good thermal conductor, suffices to generate cracks along the delaminated circumference (Figure 1d). With these ideas in mind, we next consider how the thermal properties of the support affect the propensity of PDMS wrinkled films to crack.

**Figure 1:**
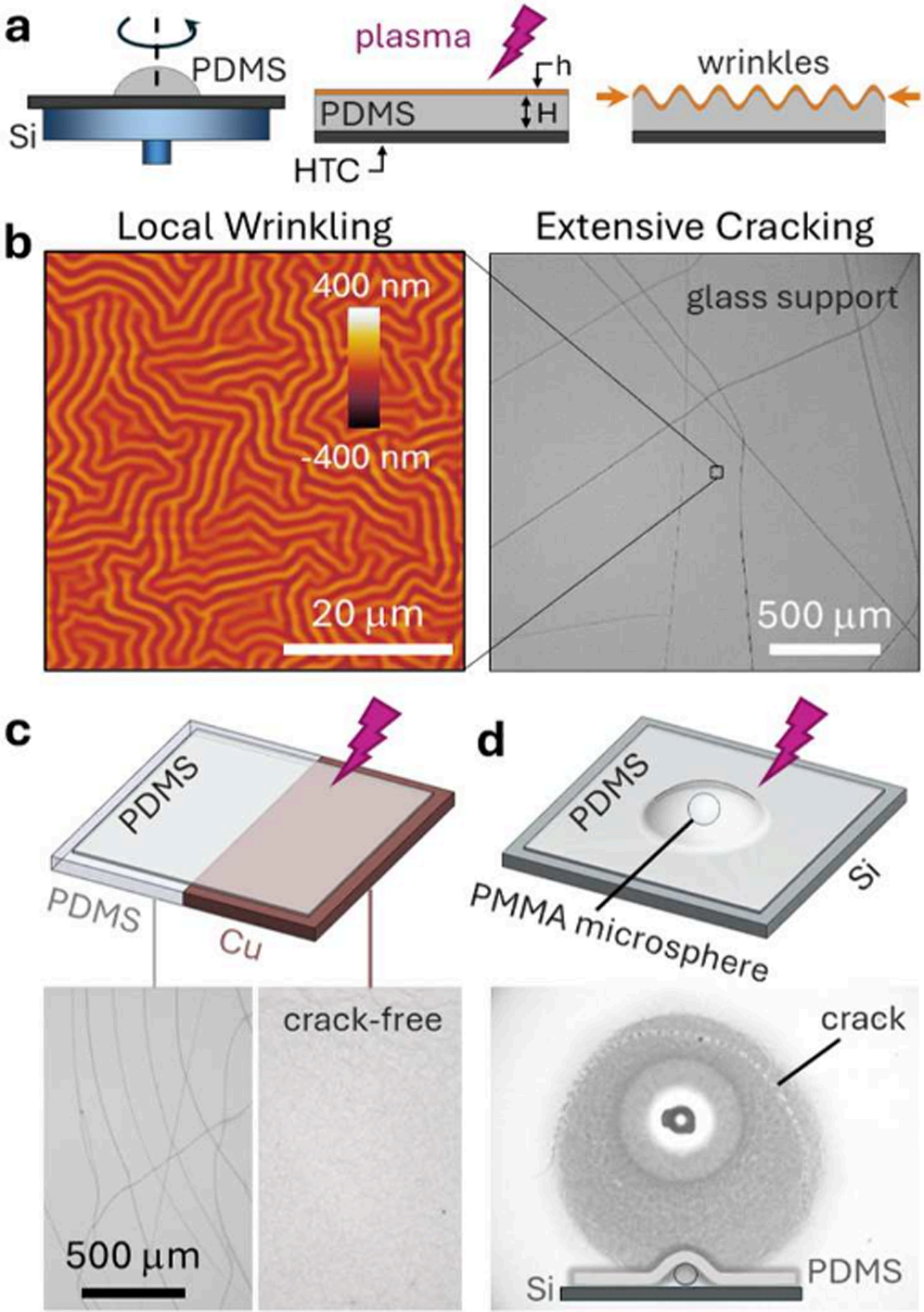
(a) Schematic of wrinkle formation by plasma oxidation of supported thin PDMS films, where HTC is heat transfer coefficient, *H* is PDMS thickness, and *h* is oxidized layer thickness. (b) Representative isotropic wrinkling at the local scale (≲ 1 mm) with extensive cracking at the micron to mm scale (*H* = 97 μm, supported on Si, placed on glass; profile ⑦ below). (c) Plasma oxidized PDMS film H = 50 μm supported across PDMS and Cu substrates. (d) PDMS film *H* = 50 μm on Si, with a PMMA microsphere (diameter 130 μm) placed in between. In all cases, O_2_ plasma at 0.2 mbar is employed, and the exposure time is 5 min in (b,c) and 10 min in (d).

Employing a good thermal conductor, Si (*K* ≈ 150 W/(m K)), as the substrate of PDMS films, we first examine the role of plasma O_2_ gas pressure *p* and exposure time on wrinkling outcomes, while monitoring the surface temperature. Figure 2a shows the decrease of wrinkling λ with *p* (from ∼6 μm to ∼500 nm for *p* = 0.05 mbar to 0.35 mbar), for PDMS *H* = 10.5 μm on Si, supported by an Al base plate (a configuration termed ①), following 1 min plasma exposure (expected due to slower kinetics of oxidation reaction^43^ with increasing *p*). Wrinkling appears generally crack-free for *p* ≥ 0.1 mbar, below which cracks form alongside wrinkles; at sufficiently low *p* (<0.05 mbar) the plasma no longer ignites. At *p* = 0.05 mbar, the temperature rises to 53 °C (Δ*T* = 31 °C), resulting in cracking alongside wrinkling; by contrast, it rises only to 29 °C (Δ*T* = 7 °C) at 0.2 mbar, yielding crack-free wrinkling instead. Analogous results are obtained for air plasma (SI Figure S2). To investigate whether prolonged plasma exposure could damage otherwise “crack-free” PDMS surfaces, films with *H* = 10.5 μm (profile ①) were exposed for 1–10 min at a fixed *p* = 0.2 mbar: while λ increases with exposure time (Figure 2c), and the temperature rises (Figure 2d) up to 58 °C during 10 min exposure (Δ*T* = 36 °C), significantly, all surfaces remain crack-free, as indicated by the green shading in Figure 2c (although Δ*T* is approximately that of 1 min at *p* = 0.05 mbar, the thermal excursion is faster at low *p*). The possible role of the cooling profile, after cessation of plasma, on crack formation was investigated by preheating PDMS films (*H* = 10.5 μm) to 75 °C and plasma oxidizing for 5 min (0.2 mbar, 99 W) before cooling down following 0.1, 50 °C/min, and Newtonian profiles to room temperature (22 °C) and held for 2 h. No cracking was observed in any case. Further, surface interactions were examined by comparing identical films supported by glass, Si, and polymeric thermal tape and PDMS, as shown in Figure 2c. No correlation between cracking and the (water) contact angle is found; instead, substrate thermal conductivity is confirmed as the governing factor (Figure 2d).

**Figure 2:**
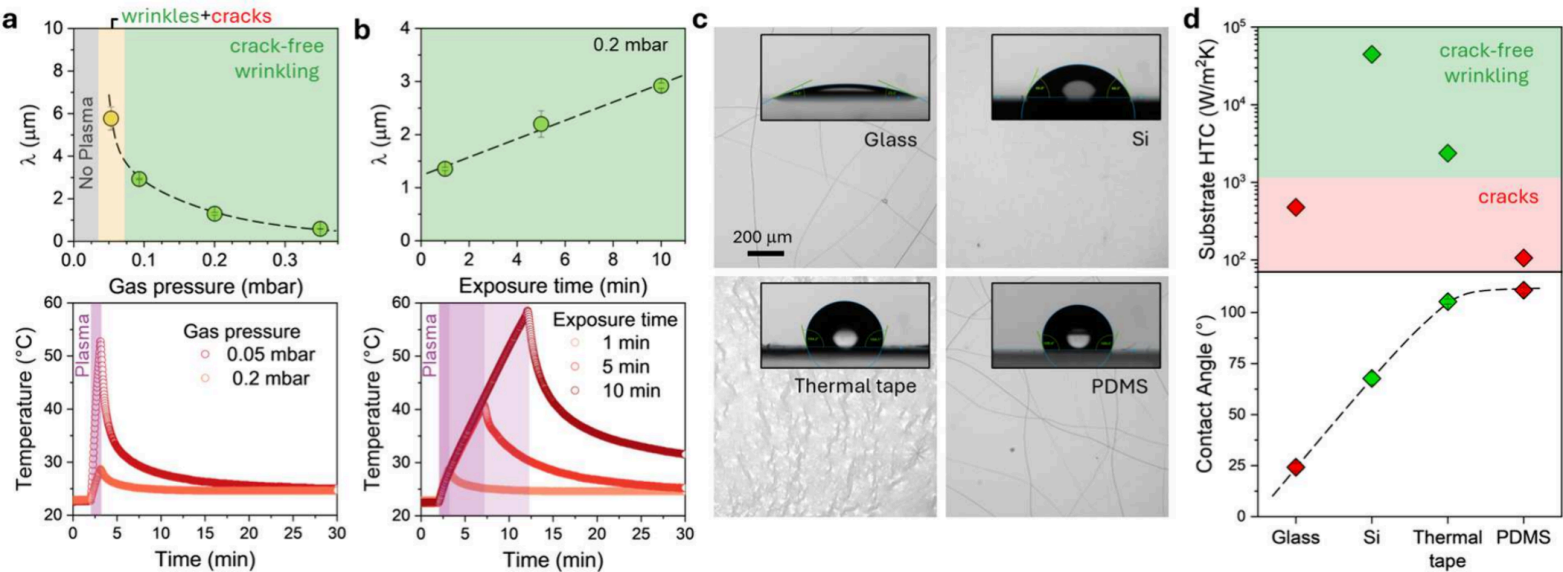
(a) Wrinkling wavelength as a function of O_2_ plasma pressure for PDMS films (*H* = 10.5 μm) placed on high HTC support (Si and Al base plate, termed ①) and plasma exposed for 1 min; corresponding thermal profiles at 0.05 and 0.2 mbar of O_2_, with 1 min plasma exposure indicated by the shaded area. (b) Wrinkling wavelength dependence on the O_2_ exposure time at 0.2 mbar for PDMS with *H* = 10.5 μm on ① substrate, and corresponding temperature profile, where the shaded areas indicate 1, 5, and 10 min exposure. (c) Optical images of PDMS films with *H* = 50 μm supported by glass, Si, thermal tape, and PDMS layers following plasma exposure (5 min, 0.2 mbar); corresponding water contact angle measurements of the neat surfaces (prior to plasma exposure). Crack-free films are obtained on Si and thermal tape, with the visible streaks in the latter corresponding to the tape itself (SI section S4). (d) Substrate HTC and water contact angle for the substrates in (c). The dashed line is a guide to the eye indicating that no correlation is found with cracking and contact angle, but instead with HTC.

Since PDMS films are generally placed atop metal, glass, and plastic supports and placed on a base plate (a “tray”, typically aluminum or glass) for plasma exposure under vacuum (Figure 3a), we estimate the heat transfer coefficient (HTC) of such layered structures as a series of resistors
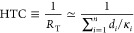
1where, in the last equality, we neglect convective terms and contact resistance between layers. The HTC is the reciprocal of the total thermal resistance, *R*_*T*_, calculated as the sum of the ratio of the material thickness, *d*_*i*_, to its thermal conductivity, κ_*i*_, for every layer *i*.^44^ The substrate HTC values are provided in Figure 3b (and tabulated in methods section, Table 1) for the profiles investigated. The highest thermal conductivity is for PDMS/Si placed on an aluminum base ① (∼10^5^ W/m^2^K), while the lowest corresponds to placing a 2 mm thick plastic coupon between the PDMS/Si and the aluminum base plate ⑨ (yielding ∼10^2^ W/m^2^K).

**Table 1:** Thermal Layer Profile and Estimated Substrate HTC^a^

Profile	Base plate	Intermediary Layer	HTC (W/(m^2^ K))
①	Al (3 mm)		42347
②	Glass (5 mm)		240
③	Al (3 mm)	Copper (1.28 mm)	37434
④	″	Brass (1.72 mm)	25355
⑤	″	S Steel (1.16 mm)	9600
⑥	″	Coverglass (370 μm)	3013
⑦	″	Glass (1.1 mm)	942
⑧	″	Glass (2.2 mm)	476
⑨	″	PDMS (2 mm)	80

a All PDMS films were supported on a silicon wafer (380 μm), included in the HTC calculation.

**Figure 3:**
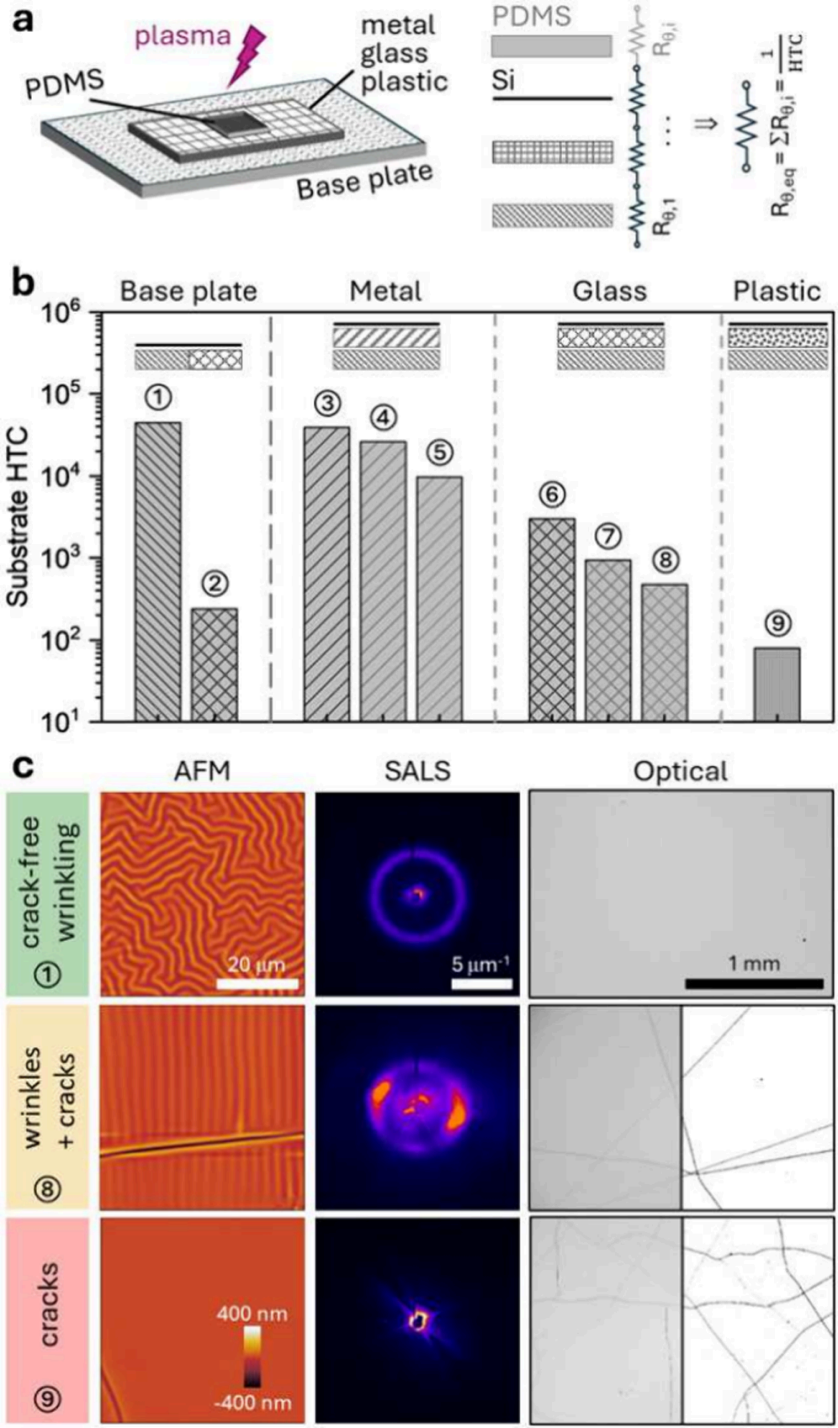
Design of heat transfer coefficient (HTC) of PDMS support fully suppresses intrinsic cracking. (a) Total thermal resistance of the substrate during PDMS plasma oxidization modeled as a number of resistors in series. (b) Estimated substrate HTC of the investigated thermal layer configurations (detailed in Table 1). (c) Representative outcomes of PDMS film plasma oxidation, observed by AFM, SALS, and optical microscopy: (top row) crack-free wrinkling (*H* = 10.5 μm, support ①); (middle row) wrinkles + cracks, imaged near a crack (*H* = 10.5 μm, support ⑧); (bottom row) cracks (*H* = 10.5 μm, support ⑨). In all cases, plasma pressure was 0.2 mbar and exposure time was 5 min . Optical images were binarized to highlight the crack density.

The clear relation between substrate HTC and film morphology, based on AFM, SALS, and optical microscopy, is shown in Figure 3c. Crack-free samples are obtained on thermally conductive supports and thin PDMS films, while extensive cracking is found in thick films or low thermal conducting supports, generally suppressing wrinkling. At intermediate conditions, wrinkles and cracks coexist, with varying density depending on HTC and plasma exposure conditions. Near cracks, wrinkling is no longer isotropic and instead becomes uniaxial, normal to the cracks, as symmetry is broken by the stress relaxation afforded by the crack. Within the crack-free region, the wrinkling amplitude remains largely unchanged at different HTC values (SI Figure S3), while increasing crack density causes the amplitude to decrease and eventually vanish, as crack formation provides a competing stress relaxation mechanism.

A quantitative map is proposed in Figure 4a, summarizing the results of 45 distinct experiments at varying PDMS film thicknesses *H* and thermal profile ①–⑨, while keeping plasma process parameters constant (40 kHz, 5 min, 0.2 mbar O_2_, 99 W). At higher *H* values (≳ 300 μm) cracking dominates, regardless of HTC; similarly, for low HTC (≲250 W/(m^2^ K)) regardless of H, all surfaces crack. A judicious combination of high enough HTC and low enough *H* provides consistently crack-free wrinkled PDMS surfaces. While the data shown apply to PDMS thin films (10:1 base:cross-linker) and fixed plasma processing conditions, the behavior is general and, for instance, increasing exposure time or decreasing pressure will decrease the “green” domain (SI Section S5). In order to estimate a threshold for the formation of crack-free bilayers during plasma exposure, we consider (i) the thermal profile of the laminate structure and its excursion during plasma processing, (ii) the residual thermal stress of the glassy skin, sequentially formed on PDMS, and (iii) its relation to the critical stress value for crack formation. We obtain the spatiotemporal thermal profile across the film *T*(*z*,*t*) by numerically solving the 1D transient heat conduction equation^44^ neglecting radiative heat losses, along the *z* axis (normal to the film surface),
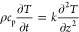
2where *k* is the thermal conductivity, ρ is density, and *c*_p_ is the specific heat capacity, and estimate the incoming heat flux at the top surface (*z* = 0) due to plasma and adjust the outgoing heat flux at the support (*z* = *H*) due to heat conduction to match experimental data (SI Figure S6c). In order to calculate the residual thermal stresses σ_f_ on the glassy skin/elastomer bilayer generated due to plasma exposure, we consider the gradual increase in the oxidized layer thickness (SI Section S6) and follow the treatment of Hernandez et al.^45^ and Tsui and Clyne,^46^ based on Stoney’s relation,^47^ for a thin film (coating) sequentially deposited on a substrate
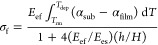
3where *E*_ef_ is taken as the effective Young’s modulus of the glassy skin, *E*_es_ is the effective Young’s modulus of the PDMS substrate, α_sub_ is the coefficient of thermal expansion of the PDMS substrate (∼3 × 10^–4^K^–1^), α_film_ is the coefficient of thermal expansion of the glassy film (∼5.5 × 10^–7^ K^–1^), *T*_rm_ is room temperature (or reference temperature), *T*_dep_ is the deposition temperature, *h* is the thickness of the skin, *H* is the thickness of the PDMS substrate, and the effective Young’s modulus is defined as *E*_e_ = *E*/(1 – ν), where *E* is the Young’s modulus and ν the Poisson ratio. Finally, we estimate the “critical stress” as^48^
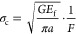
4where *G* is the strain energy release rate (0.1 J m^–2^, in line with previous work^49^), *E*_f_ is the glassy skin modulus (taken as ∼10 GPa), *F* is a dimensionless geometry factor (=1 for planar),^50^ and *a* is a precrack defect length (in-plane) , which we take as ∼100 nm based on previous work^49^ on crack formation in plasma-oxidized PDMS (SI section S7). Despite the simplicity of the model and parameter uncertainty (viz., *a*, α_f_, and *E*_f_ profile), detailed in SI Section S7, the condition σ_f_ ≤ σ_c_ captures the main features of our crack-free boundary observations in Figure 4 and SI Figure S7.

**Figure 4:**
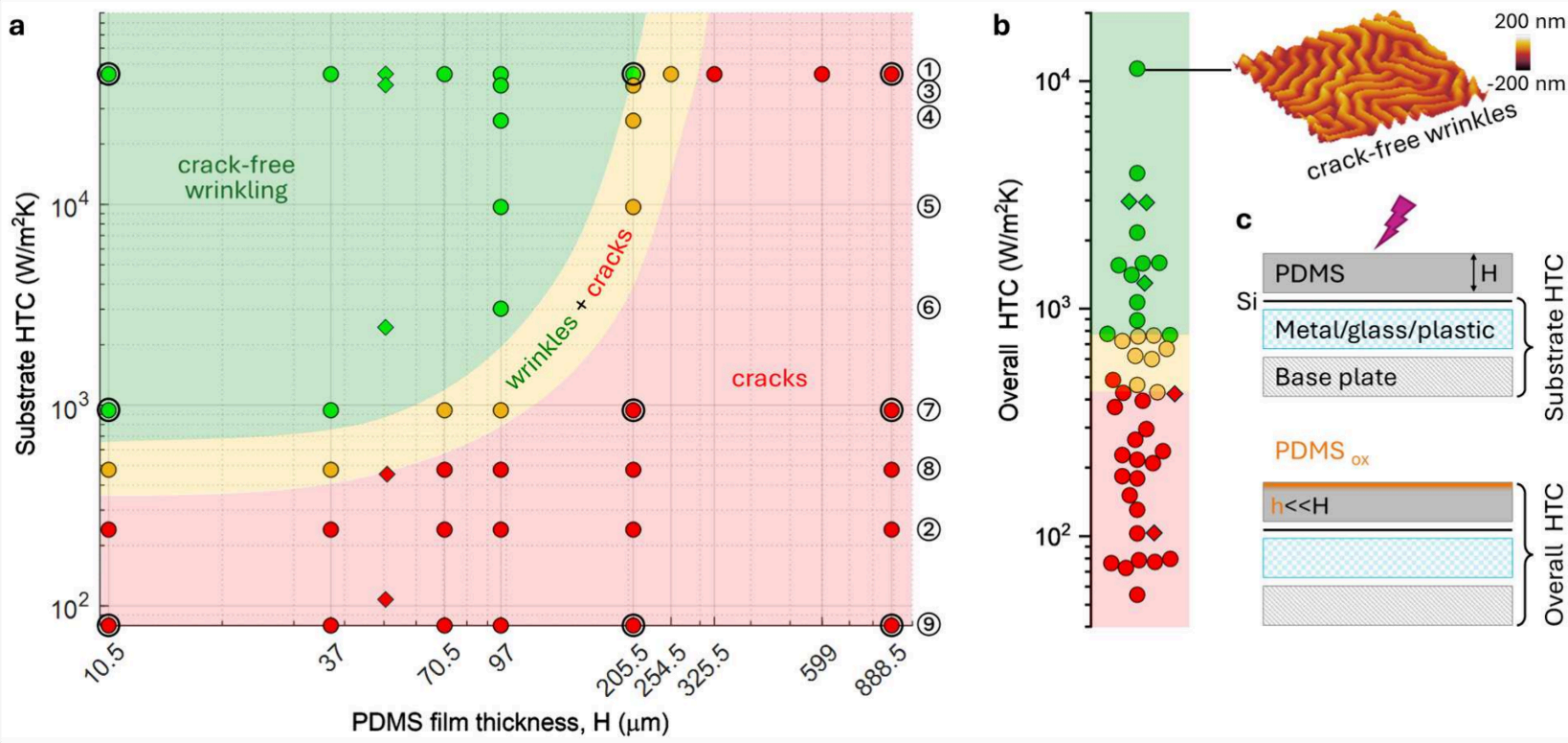
Facile approach toward crack-free PDMS wrinkled surfaces via HTC profile and a minimal model. (a) Morphology map of PDMS thin film wrinkling and cracking as a function of substrate HTC and PDMS film thickness, indicating three distinct regions of crack-free wrinkling, cracks + wrinkles, and cracks, obtained at 40 kHz plasma frequency, 0.2 mbar O_2_ gas pressure, 99 W power, and 5 min exposure time. Markers ○ correspond to wrinkled PDMS films on Si, while markers ◊ refer to films transferred onto different substrates, and selected model calculations are highlighted with concentric rings. (b) Results plotted as a function of the overall HTC, i.e. including the contribution from the PDMS film of thickness *H*, indicating crack-free wrinkling for >800 W/(m^2^ K) at these processing conditions. Points are distributed horizontally for clarity. (c) Definition of the substrate HTC and overall HTC, incorporating the resistance of the PDMS film, of thickness *H* (because glassy skin thickness *h* ≪ *H*).

We finally consider how the PDMS film *itself* might contribute to the heat transfer coefficient (HTC) and compute an *overall* HTC subsuming the PDMS layer as an additional thermal resistor with resistance depending on thickness *H*. The results are shown in Figure 4b and collapse all data into a master plot, in terms of the overall HTC: above ∼800 W/(m^2^ K) wrinkled surfaces are crack-free regardless of specific substrate HTC or PDMS film thickness. Since the glassy skin thickness (∼1–30 nm) is generally much smaller than *H* (≥10 μm, and thus negligible), we consider the entire *H* in the HTC calculation, where the PDMS film contributes as an additional resistor in series, as illustrated in gray in Figures 3a and 4c. Effective heat dissipation during and after plasma surface oxidation can thus entirely suppress the formation of surface cracks, providing a pathway for the large-scale, defect-free patterning of wrinkled surfaces. Since the surface crack density depends on process (e.g., increasing at low HTC, SI Figure S8, low plasma *p* and higher power, or subsequent thermal stress^34,48^) and evolves with time, in the present study, we have not focused on its quantification, but rather on the existence of cracks.

While the plasma exposure process causes a modest increase in temperature (Figure 2), the resulting differential thermal tensile stresses in the plane of the glassy skin layer generally result in progressive and extensive crack formation over time. Our work demonstrates that a combination of high substrate thermal conductivity and low PDMS film thickness can suppress crack formation, yielding large area patterning of PDMS with a prescribed wavelength and amplitude. The substrate thermal conductivity is effectively estimated by the heat transfer coefficient (HTC), given by the (inverse) sum of all thermal resistances of the layers supporting the PDMS film (e.g., typically glass, metal, or plastic), neglecting secondary contributions. Typically, substrate HTC > 1000 W/(m^2^ K) and PDMS film thickness *H* < 100 μm yield crack-free films. An overall HTC criterion can be defined subsuming the PDMS film itself, >800 W/(m^2^ K), yielding large-area crack-free films. While the specific criterion for the stability boundary depends on process conditions (plasma frequency, gas *p*, power, and time), shown here for 40 kHz, O_2_ 0.2 mbar, 99 W, and 5 min (Figure 4), the behavior holds generally. For instance, reducing pressure or increasing exposure time will favor crack formation by increasing environmental temperature, thereby shifting the boundaries shown in Figure 4 toward the upper left corner. Wrinkled films remain crack-free for several months, even if carefully transferred onto a different substrate after wrinkling (SI Figure S9). This suggests that effectively *stress-free* oxidized PDMS bilayer films are obtained by an appropriate design of the thermal profile. Evidently, “extrinsic” cracks can still form by subsequent mechanical tension or torsion (SI section S9), and intrinsic cracks can be selectively generated in controlled geometries (SI Figure S10) to enhance applications that harness crack-formation (e.g., gas sensing, nanofluidic manipulation, and nanofabrication^51−56^). Our findings thus provide a pathway for the full exploitation of thin film PDMS surface patterning, unlocking value from the plethora of surface patterning applications demonstrated so far.

## Methods

### PDMS Films

Polydimethylsiloxane elastomers, PDMS (Sylgard 184, Dow Corning) were prepared at 1:10 cross-linker:base ratio, degassed for 15 min under vacuum, and spun cast onto 1 cm × 1 cm silicon squares ([100] single side polish, 380 μm thick, Inseto) cut with a diamond pen knife. The wafer was first cleaned with isopropanol, and dried with compressed air. Liquid PDMS (0.2 mL) was dispensed onto the silicon support and spun coated for 1.5 min at a prescribed angular velocity (e.g., 200, 2000, and 6000 rpm yielded PDMS film thicknesses of ∼890, 40, and 10 μm), measured with a UV–vis interferometer (Filmetrics F20–UV) and a micrometer (Mitutoyo IP65). The films were then thermally cured in a 75 °C convection oven (Binder FD23) for 1 h. The static contact angle of deionized (DI) water on all substrates was measured by dispensing a 2 μL sessile drop at room temperature using a Krüss drop shape analyzer, computed using Advanced software, and averaged over at least three drops.

### Surface Plasma Oxidation

Plasma exposure was carried out with a Diener Femto 40 kHz chamber, with either oxygen or air, in order to oxidize the PDMS surface to create the bilayer and thus surface wrinkling. The gas pressure (*p*) during plasma varied to ∼0.05, 0.1, 0.2, and 0.4 mbar (corresponding gas flow rates of 0.5, 1, 5, and 10 sccm). Plasma power was fixed at *P* = 99 W, and dose is calculated as *D* ≡ *P* × *t* (e.g., *D* = 5.94 kJ, for 1 min at 99 W).^25,43^ After plasma exposure, samples were removed from the oxidation chamber and left to cool at ambient conditions on the base plate. For selected samples, the cooling profile after plasma exposure was imposed using a Linkam THMS600 thermal stage at prescribed rates from 0.1 °C/min to 50 °C/min. The temperature profile during the plasma exposure was measured with a custom-built sensor (SI Figure S10; a thermistor DS18B20 connected to an Arduino-uno, powered with a 9 V battery), at 2 s intervals and stored in a micro-SD card using an SPI adapter.

PDMS films, supported on Si wafers, were placed within the plasma chamber directly onto aluminum or glass base plates, or onto additional thermal support materials (metal, glass, plastic), as depicted in Figure 3a. Selected films were cut transferred from Si wafers to low HTC supports. The thermal conductivity of the materials employed is κ = 143 W/(m K) for aluminum alloy 5754 (Al), 1.2 W/(m K) for borosilicate glass, 398 W/(m K) for copper, 109 W/(m K) for brass, 14.4 W/(m K) for stainless steel (grade 304), 1.2 W/(m K) for borosilicate (VWR coverlips), 1 W/(m K) for thermal tape, 1.06 W/(m K) for soda lime glass (VWR glass slide), and 0.16 W/(m K) for PDMS.^57−61^ The dimensions of the base plates (Al, glass) were 25.5 cm × 8.5 cm, and the intermediate layer was cut to the dimensions of a glass slide 7.5 cm × 2.5 cm. The computed HTC values are provided in Table 1.

### Wrinkling and Cracking: Pattern Characterization

Atomic force microscopy (AFM, Bruker Innova) in tapping mode with Al-coated Si tips (MPP-11100-W, Bruker) at 0.5 Hz was employed to measure surface topography. Pattern wavelength, λ, and amplitude, *A*, were obtained from at least five distinct images (50 μm × 50 μm) from each specimen employing Gwyddion 2.62. A custom-built small-angle light scattering (SALS) apparatus, comprising a diode-pumped CrystaLaser (532 nm, 500 mW), spatial and neutral density filters, and a Hamamatsu Orca thermoelectrically cooled detector, was employed to measure surface diffraction of the PDMS specimens in reflection mode. Optical images were captured using an Olympus BX41 upright light microscope equipped with a CMOS camera (Basler acA2000-165uc) and further processed in MATLAB.

## Supporting Information Available

PDMS film preparation, air and oxygen plasma treatment, wrinkling amplitude, films on thermal tape, plasma processing conditions, contact angle measurements, glassy layer formation, heat transfer and critical stress model, crack density estimation, film aging, controlled crack formation, and temperature measurements (PDF)
