# Supplementary material for: Thermal Conduction Suppresses Cracks in PDMS Wrinkling by Plasma Oxidation
Source: Nano Lett. 2024 Dec 7;25(2):740–6. doi: 10.1021/acs.nanolett.4c05019 (PMC11741141; doi:10.1021/acs.nanolett.4c05019)
Supplement: Supplementary file 1 — nl4c05019_si_001.pdf [file nl4c05019_si_001.pdf]

Supplementary Information:

Thermal conduction suppresses cracks in PDMS wrinkling by plasma oxidation

Zain Ahmad, Begoña Parias M. R., Helen Barr and João T. Cabral*

Department of Chemical Engineering, Imperial College London, London SW7 2AZ, United Kingdom

***Email:** j.cabral@imperial.ac.uk

1 Plasma oxidation of thin PDMS films

Polydimethylsiloxane (PDMS) films of thickness H were subjected to plasma exposure through two distinct methodologies: (1) oxidation of PDMS films cured directly on silicon (Si) substrates, and (2) oxidation of cured PDMS films that were subsequently transferred onto different substrates, with various heat transfer coefficients (HTC). For the former, Si wafer squares 1 cm $\times$ 1 cm were cut using a diamond scribe, cleaned with isopropanol (IPA), and dried with compressed air. PDMS was then spin-coated onto these Si pieces at a prescribed angular velocity (Fig. S1a) to adjust H. The coated samples were cured in a convection oven, and the resulting PDMS elastomeric films on the Si substrates were exposed to plasma. Plasma oxidation was conducted by placing the PDMS-coated Si directly onto the base plate or by introducing an intermediate layer between the Si and the base plate to evaluate the influence of thermal conduction on the oxidation process. In the second method, PDMS was first spin-coated and thermally cured on a larger silicon wafer (4-inch diameter) or a glass slide (56 mm $\times$ 76 mm), both of which were cleaned and dried beforehand. After curing, the PDMS elastomer films were cut into squares (1 cm $\times$ 1 cm) using a scalpel (Fig. S1b), carefully lifted from the substrate with tweezers, and placed onto distinct substrates, including glass, PDMS, thermal tape, and Si. To minimise the entrapment of air between the film and the substrate, the corner of the square film was first brought into contact with the substrate. Any dust particles trapped beneath the film created defects, and were discarded, except in experiments discussed in Section 8.

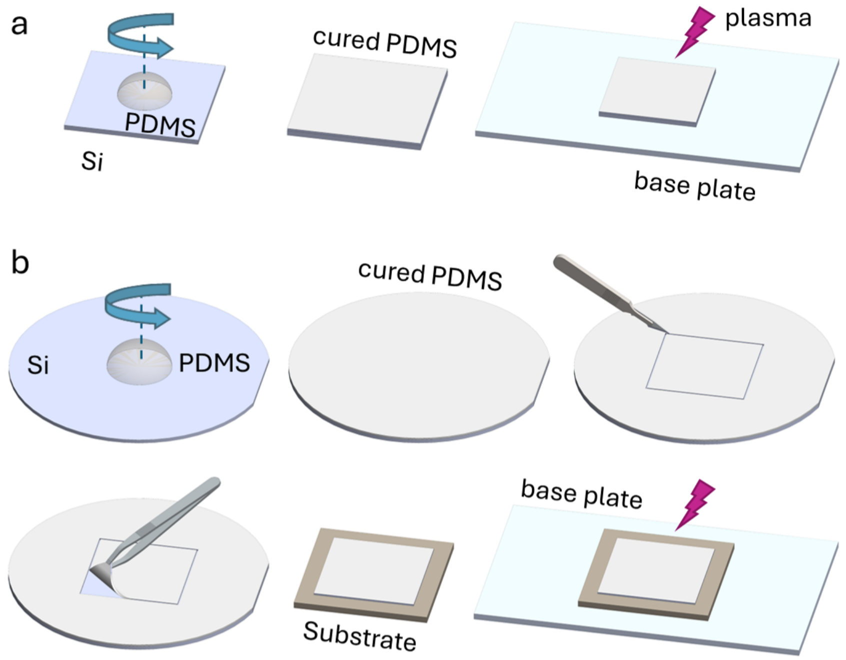

Figure S1 Schematic of plasma oxidation of PDMS films. a) PDMS spun coated on square Si wafer (1 cm $\times$ 1 cm) is thermally cured and exposed to plasma while being placed on a base plate in plasma chamber. b) PDMS spun coated on a Si wafer (4-inch diameter) is cured and scored with a scalpel. The film is lifted, placed on another substrate and then exposed to plasma.

2 Wrinkling and crack-formation with air and oxygen plasma

In order to examine the generality of our findings, we investigate the wrinkling and crack-formation outcomes of plasma exposure with air, in addition to oxygen (reported in the main paper). We select H=10.5 μm and thermal profile ① (film supported on Si wafter/Al base plate), P=99W and 1 min exposure, and varying gas pressures p. At very low pressure, $p < 0.04$ mbar, the plasma does not ignite and, evidently, no wrinkling or crack formation is observed. At p =0.05 mbar wrinkles and cracks are observed upon plasma exposure to both gases, and λ and A are highest (Fig. S2). Upon increasing p, λ and A decrease monotonically and cracks are suppressed in both cases. Previous work by Fritz and Owen examined the 'onset of microcracks' upon PDMS plasma exposure with four gases, qualitatively ranked Ar>O_2>N_2>He in terms of the "most intense plasma treatment", i.e. Ar being the most intense and He the least.[1] The similarity of our conclusions for O_2 and air provide confirmation that our findings are robust to the most common plasma operation gases, and which are amongst the most 'intense'.

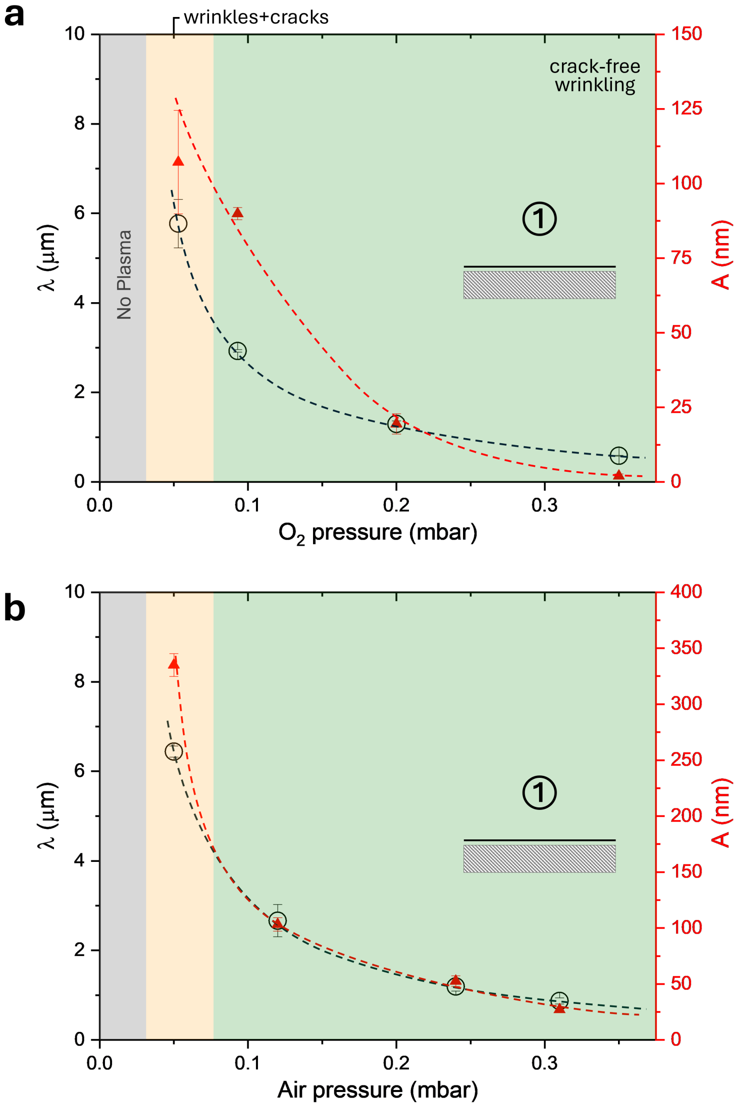

Figure S2 Wrinkling wavelength λ and amplitude A experimentally measured as a function of gas pressure, for (a) O_2 and (b) air. In all experiments, PDMS film thcikness was H=10.5 μm, plasma power was P=99 W, and exposure time 1 min, and configuration ① (i.e., the highest thermal conduction with an Al base plate and Si wafer).

3 Wrinkling amplitude vs substrate HTC

It is known that the formation of cracks leads to localised stress relaxation, particularly in the in-plane tensile direction, as the material no longer needs to stretch across the cracked region. This redistribution of stress impacts out-of-plane deformations through Poisson's effect, often causing a slight surface rise near the crack edges.[2] Additionally, the release of strain energy at the crack site reduces the energy available for driving wrinkle formation in adjacent regions. Consequently, as cracks develop, the amplitude of nearby wrinkles decreases, even as their wavelength remains unchanged. Repeated cycles of cracking and relaxation further diminish the wrinkle amplitude, as observed in experimental studies.[3,4] To determine whether the wrinkle amplitude in the crack-free wrinkling zone is influenced by the heat transfer coefficient (HTC) of the substrate, AFM scans were performed on plasma-oxidised PDMS films ($H = 10.5$ μm). The films were oxidised under plasma conditions (5 min, 99 W, 0.2 mbar O_2) and placed on two substrates with distinct HTC values: 42.3×10^{-3} W/m^{2}K and 942 W/m^{2}K, corresponding to configurations ① and ⑦, respectively (Fig. S3). Wrinkle dimensions were analyzed by generating cross-sectional profiles from the AFM images using *Gwyddion* software. Cross-sectional lines were drawn perpendicular to the direction of the wrinkles to ensure accurate measurement of the wrinkle amplitude. The line scans reveal that the wrinkle amplitude, $A \approx 100$ nm, is unaffected by the HTC values of the substrates. This indicates that within the crack-free wrinkling zone, the wrinkle amplitude is independent of the substrate's heat transfer properties.

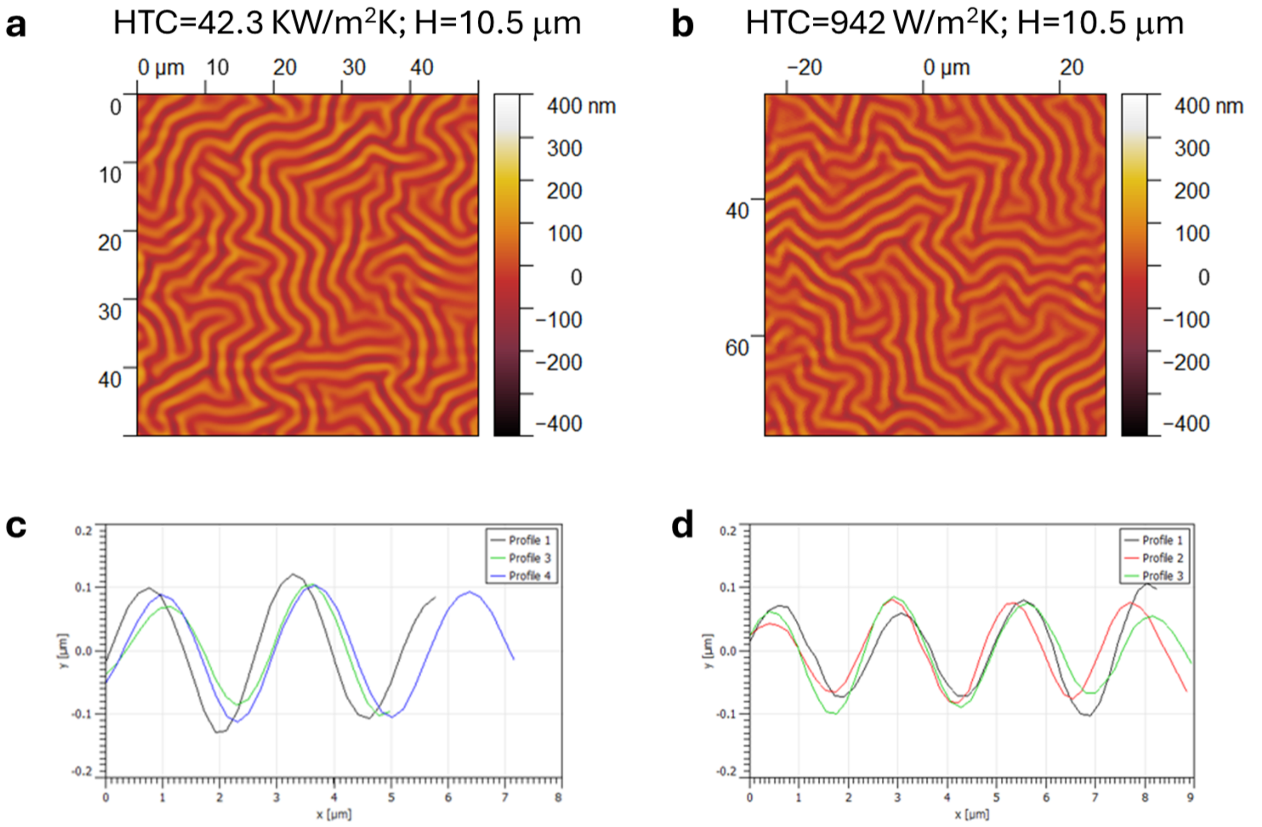

Figure S3 AFM scans of plasma-oxidised PDMS film (H=10.5 μm) treated for 5 min under 99 W power with O_2 gas at 0.2 mbar. (a) Surface profile for configuration ① and (b) for configuration ⑦. (c) and (d) show cross-sectional profiles taken across different regions of the AFM scans in (a) and (b), respectively.

4 Crack-free wrinkling on PDMS film on thermal tape

A PDMS film with a thickness H = 50 μm was placed on a polytetrafluoroethylene (PTFE)-based thermal tape and exposed to O_2 plasma for 5 min at p = 0.2 mbar. This treatment resulted in the formation of crack-free wrinkles on the PDMS surface. Although the optical images presented in Fig. 2c and Fig. S4a appear to show an underlying pattern or cracks on the surface, these correspond to the texture of the tape surface, and the wrinkled PDMS films are indeed crack free (Fig. S4b). Such patterns are are not observed when the PDMS film is transferred onto silicon, glass, or PDMS substrates.

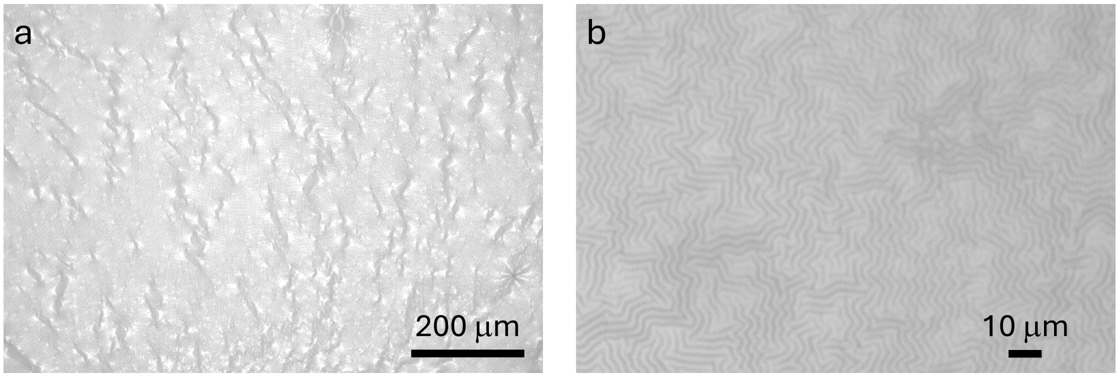

Figure S4 Optical images at two magnifications of a PDMS film (H=50 μm) supported on a PTFE-based thermal tape following exposure to plasma (5 min, 0.2 mbar).

5 Morphology map boundaries: effect of processing conditions

While the overall behaviour is general, the specific boundaries of the morphology map shown in Fig. 4 of the main paper depend on plasma processing conditions, such as gas pressure or exposure time. In order to illustrate this dependence, PDMS films with H=10.5 μm were subjected to thermal profile ⑦ and exposed to plasma at different gas pressures and exposure times, where one parameter was varied keeping the other constant. For a constant exposure time of 5 min, the morphology outcome transitioned progressively from wrinkles+cracks to crack-free wrinkles upon increasing O_2 pressure. Upon increasing time at constant pressure p =0.2 mbar, the surface morphology evolved from wrinkling to wrinkles+cracks, and eventually to cracks only.

Table S1 Outcomes from the plasma oxidation of thin PDMS film for varying gas pressure and exposure time.

Profile	PDMS thickness (μm)	Pressure (mbar)	Exposure time (min)	Outcome
⑦	97	0.057	5	wrinkles+cracks
⑦	97	0.21	5	wrinkles+cracks
⑦	97	0.35	5	wrinkles
⑦	97	0.21	1	(below ε_c)
⑦	97	0.21	1.5	wrinkles
⑦	97	0.21	2.5	wrinkles+cracks
⑦	97	0.21	5	wrinkles+cracks
⑦	97	0.21	10	cracks

6 Relative process timescales for glassy layer formation

Plasma treatment of PDMS renders the surface hydrophilic. Owen and Smith[5] reported the X-ray photoelectron spectroscopy (XPS) surface analysis of plasma oxidised PDMS, observing changes in the elemental surface composition even after 1 s of plasma exposure, and an increase in atomic composition of oxygen with longer exposure times. Under standard O_2 plasma oxidation conditions, at 99W and p=0.2 mbar, we find that full wettability to water is obtained after $<$ 5 s of plasma exposure (Figure S5). The formation and front propagation of the glassy skin follows log-time kinetics over min timescales, determined from X-ray reflectometry analysis and frontal propagation model simulations (*viz.* the glassy thickness increases from from $<$ 5 to $\sim$60 nm within $\sim$1-100 kJ plasma doses).[6–11] By contrast, the thermal excursion of the film surface is approximately linear with time, over timescales of several minutes. Drawing from both our experimental findings and previous work, we infer that the glassy layer begins to form within $\sim$1 s, while thermal excursion occurs over much longer timescales.

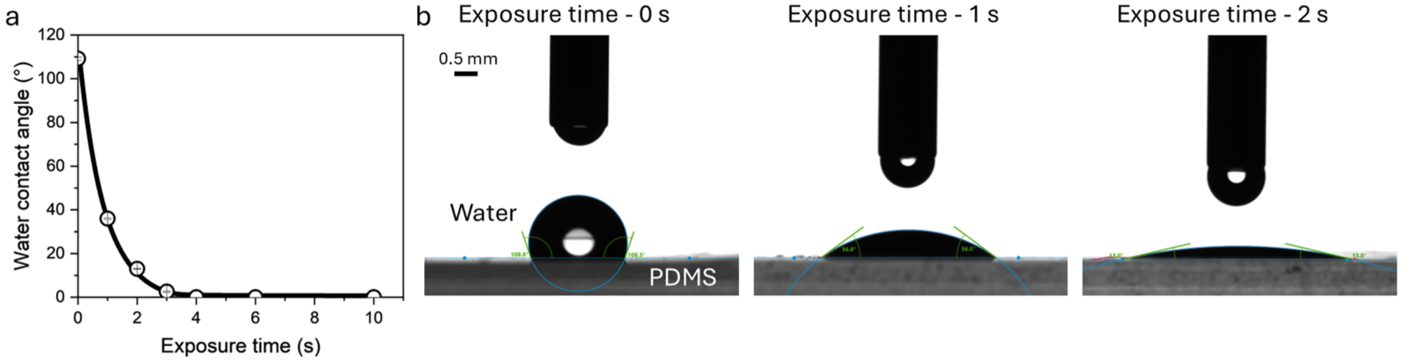

Figure S5 a) Measured water contact angles for PDMS exposed to plasma (99 W, 0.2 mbar) at varying exposure times 0-10 s. b) Optical images of the water contact angle for unoxidised PDMS (exposure time 0 s), and oxidised PDMS (exposure times 1 s and 2 s).

7 A minimal model to estimate the onset of crack formation

The conditions at which plasma oxidised samples crack can be estimated by evaluating the thermal stresses generated in the bilayer structure (glassy film on elastomer substrate) upon plasma exposure, and comparing these with the threshold stress value, characteristic of the material. Thermal stresses higher than the given threshold stress, the surface is expected to crack (forming 'intrinsic' cracks, regardless of additional mechanical or environmental stresses). This threshold value of stress, or 'critical stress', is related to the so-called critical stress intensity factor, or fracture toughness, describing the resistance to fracture of a given material. Following the treatment of Tooley et al.[12], we write the critical stress intensity factor as $K_c = \sigma_c \sqrt{\pi a} \cdot F$, where σ_c is the critical stress, a is the pre-crack defect length, and F is the dimensionless geometry factor.[13] In short, K_c characterises the stress near a crack. Given the difficulty of experimentally measuring the value of K_c for the oxidised skin layer, with thickness $\sim$10 nm, we seek to infer it from the strain energy release rate, $G = K_c^2/E_f$, where E_f is the modulus of the glassy skin.[13] Rearranging the relation above, results in a critical stress:

$$\sigma_c = \sqrt{\frac{GE_f}{\pi a}} \cdot \frac{1}{F} \tag{S1}$$

The value of G for the plasma oxidised layer from previous reports has been reported[2] to fall within $0.1-0.3$ Jm^{-2}. The modulus of the glassy layer is considered to be 10 GPa and to account for uncertainty additional $\pm$2 GPa is incorporated in the calculations. The length of the pre-crack (a) is assumed to be $\sim$100 nm. Previous research on crack formation in plasma-oxidised PDMS by Mills et al.[2] analysed nanocrack profiles using SEM, AFM, and numerical simulations, reporting crack widths around 100 nm and larger, with significantly longer crack lengths. Their findings, combined with observations of thermal stress accumulation and surface nonuniformities, suggest that a pre-crack defect length of 100 nm represents the smallest dimension reported and serves as a reasonable starting point for understanding the crack formation process. The dimensionless geometry factor (F) for planar geometry can be assumed to be 1.[13] The evaluated critical stress, σ_c, is shown in Fig. S6a, b as dotted lines, where the shading corresponds to a range in modulus values.

Separately, the residual thermal stress σ_f within a thin film sequentially deposited on a substrate can be estimated using Tsui and Clyne's model[14] based on Stoney's relation,[15] following the treatment of Hernandez et al.[16] yielding

$$\sigma_f = \frac{\left[E_{ef}\int_{T_{\text{rm}}}^{T_{\text{dep}}}(\alpha_{\text{sub}} - \alpha_{\text{film}})\,dT\right]}{\left[1+4\left(\frac{E_{ef}}{E_{es}}\right)\left(\frac{h}{H}\right)\right]} \tag{S2}$$

where E_{ef} is the effective Young's modulus of the film, E_{es} is the effective Young's modulus of the substrate, α_{sub} is the coefficient of thermal expansion of the PDMS substrate ($\sim$3 10^{-4}K^{-1}),[17,18] α_{film} is the coefficient of thermal expansion of the glassy film ($\sim$5.5 10^{-7}K^{-1}),[12] T_{rm} is room temperature (or reference temperature), T_{dep} is the deposition temperature, h is the thickness of the film, and H is thickness of the substrate. The effective Young's modulus is given as $E_e = E/((1-\nu)$, where E is the Young's modulus and ν is the Poisson ratio. Although the above model was developed to predict residual thermal stress in progressively deposited coating, we consider the gradual glassy skin formation thorough plasma exposure as a coating process. We now seek to estimate the thermal profile of the laminate structure, for which we write the governing equation for 1D transient heat conduction[19] along the z axis

$$\rho c_p \frac{\partial T}{\partial t} = k\frac{\partial^2 T}{\partial z^2} \tag{S3}$$

where T is the temperature (K), t is the time (s), z is the spatial coordinate (m) normal to film surface, k is the thermal conductivity (Wm^{-1}K^{-1}), ρ is density (kgm^{-3}), and c_p is the specific heat capacity (Jkg^{-1}K^{-1}). The heat propagation in just one direction in absence of any radiative heat loss is a simplified analysis for temperature evolution across the sample. Using the finite difference method, we discretise the equation in both time and space, and T_i^n represents the temperature at node i and time step n, Δt be the time step, and Δz be the spatial step. For internal nodes, we apply the central difference method in space and the forward difference method in time, yielding:

$$\rho c_p \frac{T_i^{n+1} - T_i^n}{\Delta t} = k\frac{T_{i+1}^n - 2T_i^n + T_{i-1}^n}{(\Delta z)^2} \tag{S4}$$

and rearranging to solve for T_i^{n+1},

$$T_i^{n+1} = T_i^n + \alpha\frac{\Delta t}{(\Delta z)^2}\left(T_{i+1}^n - 2T_i^n + T_{i-1}^n\right) \tag{S5}$$

where $\alpha = \frac{k}{\rho c_p}$ is the thermal diffusivity. At the top surface, with an incoming heat flux, solving for T_0^{n+1}, we obtain

$$T_0^{n+1} = T_0^n + 2\alpha\frac{\Delta t}{(\Delta z)^2}\left(T_1^n - T_0^n\right) + \frac{2q_{\text{in}}\Delta t}{\rho c_p \Delta z} \tag{S6}$$

and at the bottom surface, solving for T_N^{n+1}:

$$T_N^{n+1} = T_N^n + 2\alpha\frac{\Delta t}{(\Delta z)^2}\left(T_{N-1}^n - T_N^n\right) - \frac{2q_{\text{out}}\Delta t}{\rho c_p \Delta z} \tag{S7}$$

Employing this minimal model for the thermal stresses and temperature profile evolution of a supported PDMS film, we obtain the estimates shown in Fig. 4. For a thermal substrate ①, the critical stress σ_c and film stress σ_f are estimated to intersect at $\sim$ 200 nm, which agree with our observation at those process conditions, as shown in Fig. S6a. The shaded areas indicate the (significant) uncertainties and assumptions in estimating σ_f associated with the thermal excursion, modulus of the glassy layer (E_f $\sim$10$\pm$2 GPa, which is moreover known to exhibit a gradient[1,7,20]); as well as σ_c, related primarily to the length of the pre-crack defect, which is estimated to range from $\sim$ 10 nm (from nanoparticle permeation[12]) to $\sim$100 nm. These were treated as fitting parameters within physically reasonable boundaries to test the applicability of the model, rather than to predict an exact stability boundary for crack-formation from first principles. At fixed H=200 μm, Fig. S6b establishes the relative magnitudes of σ_f and σ_c as a function of temperature differential experienced during plasma exposure; cracking is expected at $\Delta T \gtrsim 25$°C.

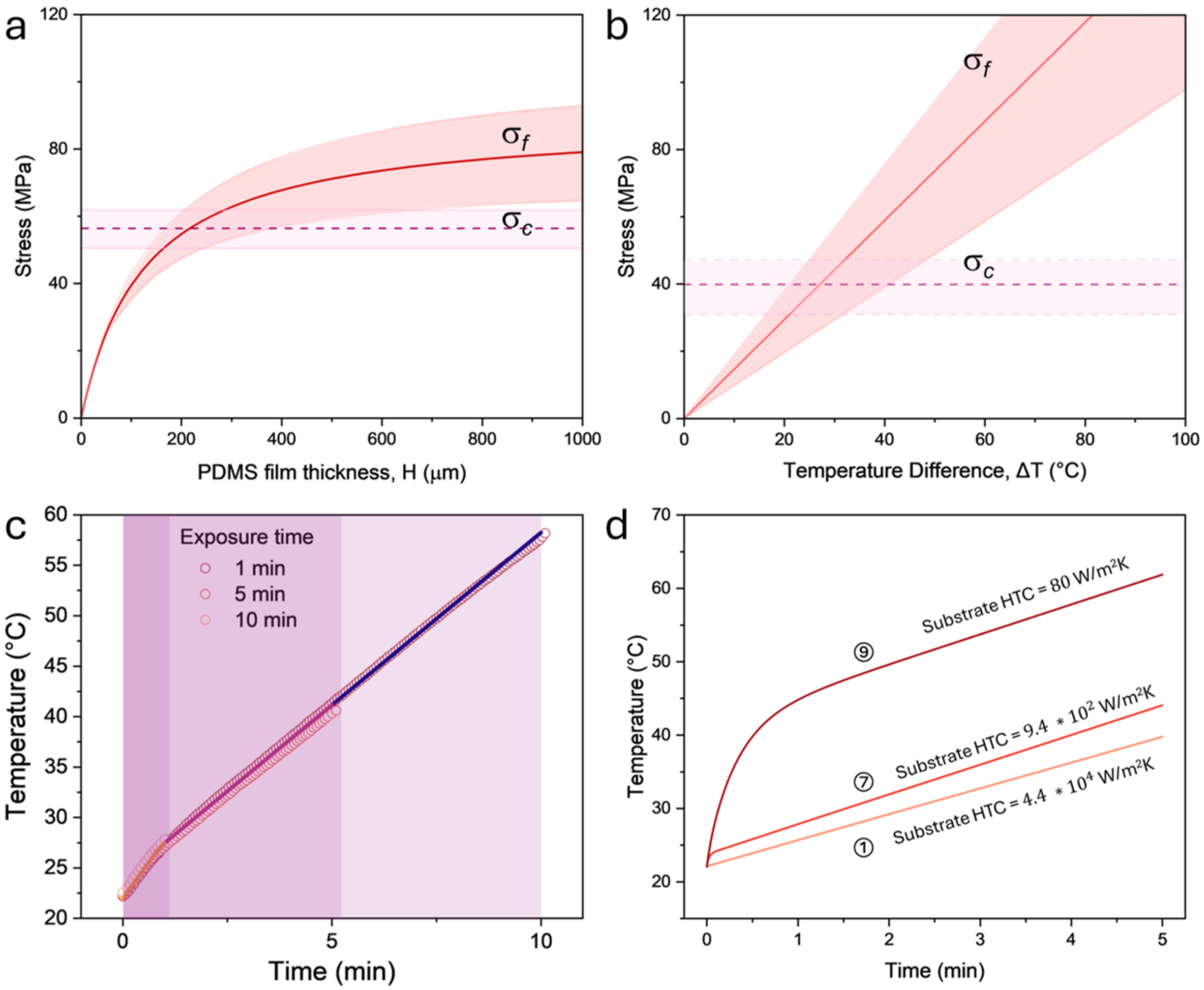

Figure S6 a) Calculated thermal stress, σ_f, as function of PDMS film thickness for $T_{\text{dep}} = 45$ °C shown as solid line, corresponding to ① film structure (P = 99 W, 5 min, p = 0.2mbar, and the critical stress for fracture, σ_c, as dashed line. The shaded region accounts for a range of uncertainties in modulus of glassy layerE_f and pre-crack length detailed in text. The thermal stress, σ_f, intersects critical fracture stress, σ_c, at $\sim$200 μm. b) σ_f and σ_c as a function of temperature difference for ① film structure and PDMS film thickness, H=10.5 μm. c) Calculated temperature profile (solid line) from transient 1D heat transfer model upon 1, 5 and 10 min of plasma exposure based on the experimentally observed temperature values (circles, reproduced from main paper Fig. 2d). Temperature evolution at the top surface of PDMS, for fil thickness, H=10.5 μm exposed to plasma for 5min at different sample profiles ①, ⑦ and ⑨, corresponding to substrate HTC values 4400 W/m^{2}K, 940 W/m^{2}K, and 80 W/m^{2}K respectively.

The above 1D transient heat transfer equations were utilised to obtain the temperature vs time relation for sample profile ①. The boundary condition at the top node experiences an incoming heat flux, which is taken as $q_{\mathrm{in}} = P\eta/A$, where P is plasma power, η is the efficiency ($\sim 40\%$) and A is the area of the base plate. The outgoing heat flux (q_{in}) was adjusted to overlap the simulated thermal profile with the experimentally observed temperature profile. The measured temperature recordings (reproduced from Fig. 2d of the main paper) for O_2 plasma exposure times 1, 5 and 10 min, at p=0.2 mbar and P=99W and computed profiles for a representative surface (Al plate ①) are shown in Fig. S6c. The aforementioned boundary conditions were set to simulate the temperature variations at the top of PDMS (H=10.5 μm) for three different thermal profiles ①, ⑦ and ⑨. Fig. S6d illustrates how the temperature on surface depends on the HTC of the substrate, with high thermal conductors evidently leading to more modest temperature increases (① corresponds to the bottom curve). Additionally, from the heat transfer analysis, it is observed that the difference in temperature between the top of PDMS film and the Al base plate after 5 min of plasma exposure for sample profile ① is $\sim$0.2 °C, whereas for sample profile ⑨ is $\sim$22 °C. The temperature profile is then utilised in providing an estimate for the thermal stresses experienced during plasma oxidation from eq. S2.

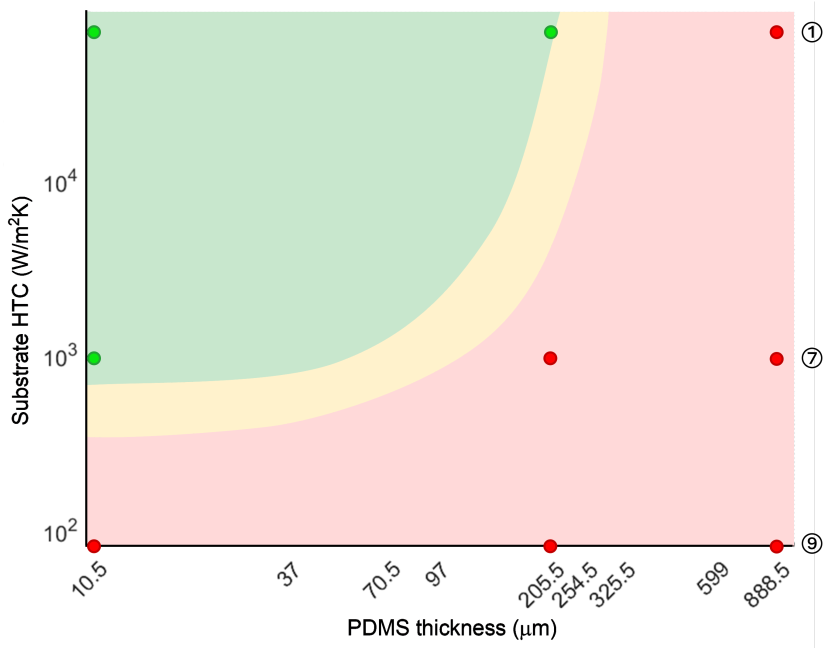

Figure S7 Computed morphology map for illustrative HTC values 4400 W/m^{2}K ①, 940 W/m^{2}K⑦, and 80 W/m^{2}K ⑨, and selected film thickness H, corroborating the experimental map reported in Fig. 4a of the main paper. In these calculations, we have adjusted model parameters, including the pre-crack length a, and estimated skin modulus E_f and specific ΔT for the laminate structure to refine the agreement with experiment (see text for details).

Overall, these results enable us to estimate the boundaries shown in Fig. S7, which are compatible with our experimental observations (noting the significant caveats and assumptions above), generally corroborating the emergence of crack-free surfaces at high HTC and lower H. The exact location of the boundaries depend on plasma process parameters and model assumptions. This minimal framework allows us, nonetheless, to elucidate the overall physical behaviour and support our design conclusions.

8 Crack density estimation from optical microscopy

To estimate the crack density in samples exhibiting cracks (red) or wrinkles+cracks (orange), selected optical microscopy images were processed using MATLAB, a binary threshold applied, and the areal coverage of cracks computed across representative imaged areas and specimen repeats. The crack density increases with time, with mechanical handling of the specimen, and additional environmental stresses (e.g., fluctuations in temperature, humidity and gaseous environment) and, therefore, our paper focuses on establishing a morphology diagram instead of rigorously quantifying crack density. Nonetheless, Fig. S8 illustrates the crack density obtained in PDMS films with H=10.5 μm, exposed to O_2 plasma at p =0.2 mbar, for 5 min placed on distinct thermal substrates. For thermal profile ① and ⑦ crack-free wrinkles emerge, cracks and wrinkles are observed for ⑧ and only cracks formed for thermal profile ② and ⑨. The substrate HTC values for ⑧, ② and ⑨ are 477 W/m^{2}K, 240 W/m^{2}K and 80 W/m^{2}K respectively. For a fixed film thickness, the crack density increased with decreasing substrate HTC at otherwise identical conditions. There was a significant jump in the density from profile ⑧ to profile ② ($\simeq$ 5%) compared to that from ② to ⑨ ($\simeq$ 2%), associated with the change in outcome from wrinkles+cracks to cracks only.

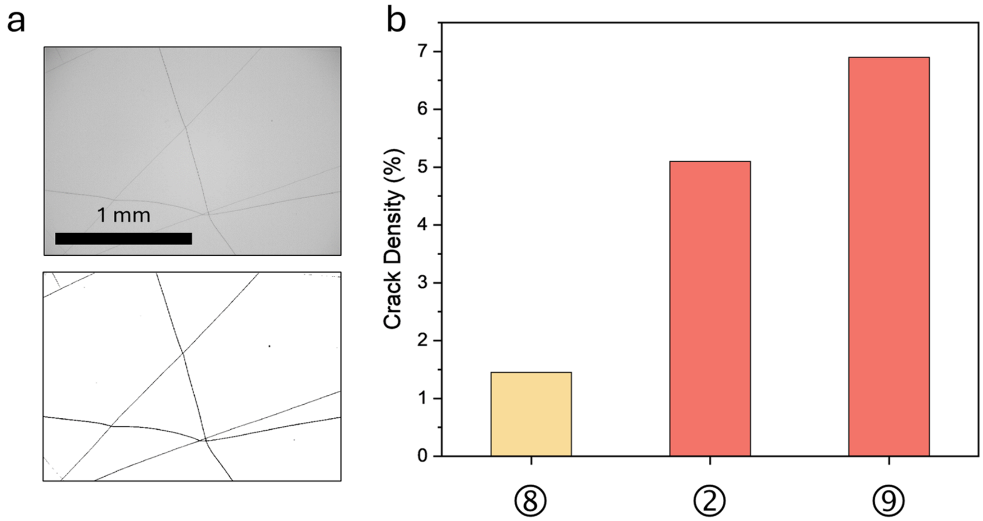

Figure S8 a) Optical image of cracks (H = 10.5 μm, support ⑧) and binarised image employed to estimate crack density. b) Crack density estimated for H=10.5μm films, exposed to O_2 plasma at p =0.2 mbar, for 5 min, subjected to thermal profile ⑧, ② and ⑨.

9 Ageing and handling of plasma oxidised PDMS films

To investigate possible aging processes and slow emergence of cracks on wrinkled PDMS films, samples were monitored over a two-month period. Specimens included those spin-coated and wrinkled directly on Si, and those subsequently transferred onto low HTC substrates.

A PDMS film with a thickness of 50 μm was initially placed on a Si wafer and exposed to O_2 plasma for 5 min at p = 0.2 mbar, resulting in the formation of crack-free wrinkles. After two weeks, the film was lifted from the substrate using tweezers and redeposited on another Si wafer for imaging. After this transfer, the film remained wrinkled and crack-free (Fig. S9a), although in some sections undulations along the direction of redeposition appeared (interpreted as 1D strain imposed during lifting). Sections that undergo significant stretching and bending during transfer exhibit micro-cracks on the surface, thus 'extrinsic' cracks. The visual appearance and structural (diffractive) colour[21–25] of such films is evidently impacted by crack-formation.

A separate experiment was conducted with a PDMS film cured on Si with H = 10.5 μm, which was also exposed to plasma for 5 min at 0.2 mbar. This treatment similarly resulted in the formation of crack-free wrinkles. The wrinkled surface was monitored over several months to determine if any cracks would develop over time. The optical image in Fig. S9b, taken two months after plasma exposure suggests that films are effectively stress-free, as no cracks form after prolonged ageing.

To differentiate between intrinsic and extrinsic cracks and to replicate crack formation during the transfer process due to bending and stretching, a crack-free wrinkled PDMS film (H=10.5 μm) on Si, produced by plasma exposure (5 min, 0.2 mbar), was subjected to uniaxial stretching one day after plasma oxidation (Fig. S9c) using a strain stage. After slowly releasing the applied stress, the resulting film exhibited micro-cracks on its surface, as shown in Fig. S9d.

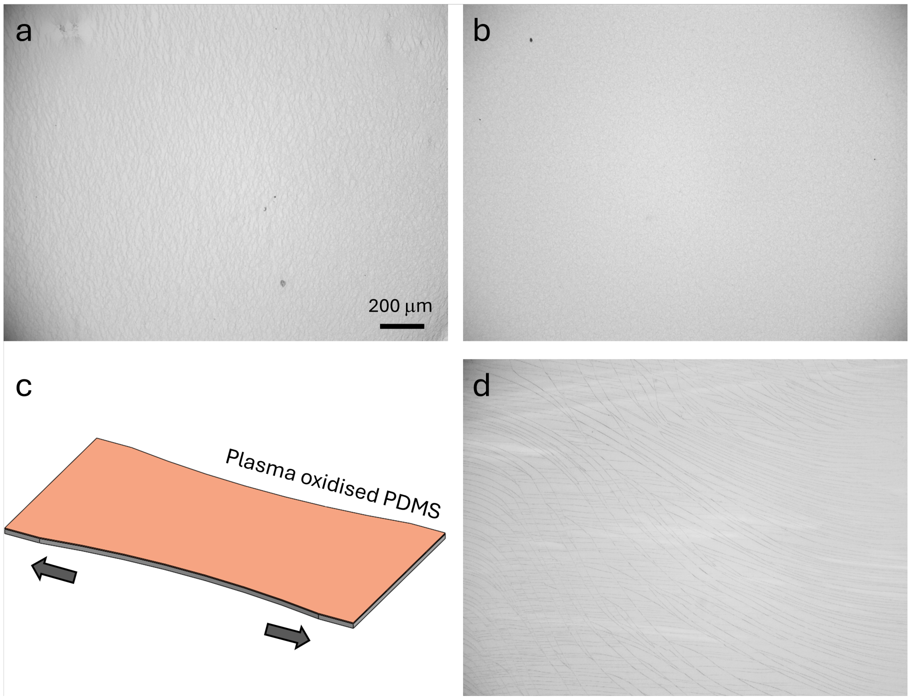

Figure S9 a) Optical images of (a) plasma oxidised film (H = 50 μm, plasma for 5 min at 0.2 mbar) redeposited on silicon wafer after 2 weeks and (b) PDMS film on Si after 2 months of plasma exposure (H = 10.5 μm, plasma for 5 min at 0.2 mbar). c) Schematic of the uniaxial stretching of plasma oxidised PDMS. d) Optical image of oxidised PDMS film (H = 50 μm, plasma for 5 min at 0.2 mbar) subjected to uniaxial stretch (ε=20%) and released after.

10 Controlled crack formation

During the transfer and re-deposition of PDMS films onto a substrate, air can become trapped, due to a crease or a particle (e.g., dust, fibre) between the substrate and the PDMS film, leading to the formation of dome-like structures. Such domes deform under plasma oxidation, as a result of the vacuum and heat generated, during glassy skin formation. Upon cooling to ambient conditions, Fig. S10a illustrates circular cracks observed in an oxidised PDMS film (H=50μm, 5 min, 0.2 mbar) that was placed on thermal tape with a dust particle/fibre trapped beneath it. These cracks are primarily attributed to the poor heat conduction caused by the presence of the air pocket. Additionally, the wrinkles align perpendicularly to the contact line of the dome and, inside the circular region, isotropic wrinkles are accompanied with cracks.

Understanding the impact of heat conduction on crack formation enables the generation of cracks in controlled geometries. We demonstrate controlled crack formation along parallel strips using a substrate patterned with SU8 photoresist, with SU8 features with a width of 500 μm and a height of $\sim$100 μm on Si. A PDMS film with a thickness of 50 μm was placed on the patterned substrate and exposed to plasma for 5 min at 0.2 mbar. Cracks formed selectively in regions where the PDMS film was not in contact with the substrate, i.e. within channels (Fig. S10b).

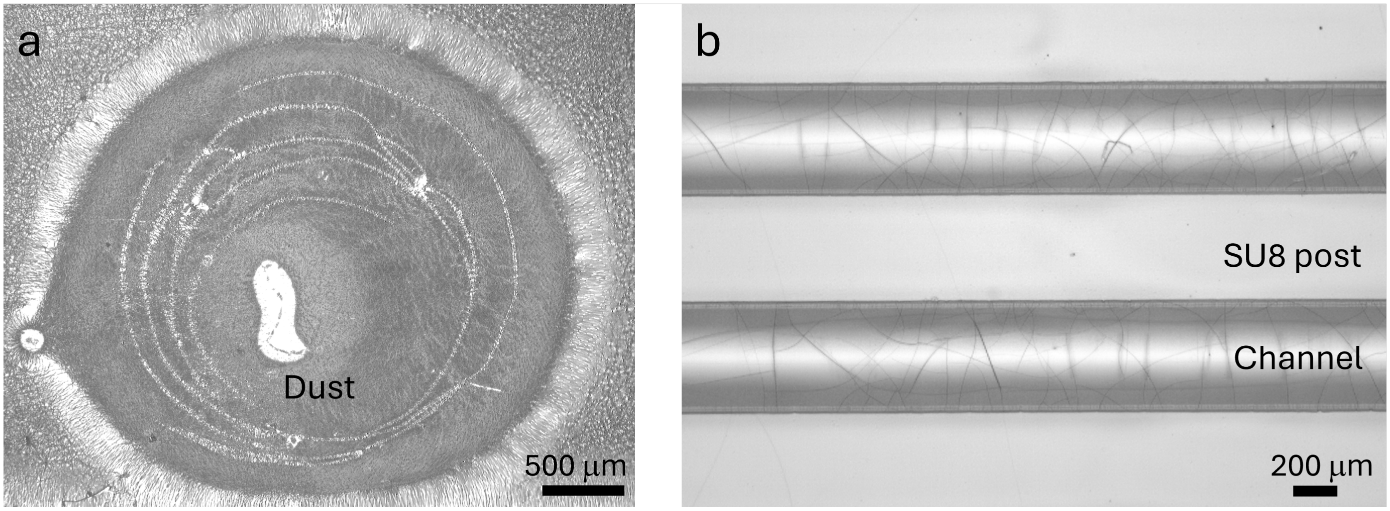

Figure S10 Optical images of (a) circular crack formation around a dust particle/fibre trapped beneath oxidised PDMS film (H=50μm, 5 min, 0.2 mbar) placed on thermal tape, and (b) PDMS film (H=50 μm) across parallel SU8 posts (500 μm wide and $\sim$100 μm high) following plasma exposure (5 min, 0.2 mbar).

11 Temperature measurement during plasma processing

In order to track the temperature profile during plasma exposure, a wireless Arduino-based temperature sensor, able to measure and store temperature values, was designed and assembled. Arduino-uno, micro-controller based on ATmega328P, was attached with prototyping shield breadboard 5V 1A 2.54mm SYB-170 offering extended mounting options. Micro SD SPI storage board TF memory card adapter shield module 3.3 5V was the interface between SD card and arduino connected with the help of jumper wires on bread board(Fig S11a). The temperature sensor probe, thermistor DS18B20, was interfaced with Arduino by adding a 4.7K resistor. Arduino IDE 2.1.1 was used to provide instructions to this custom sensor by utilising device specific libraries: SPI.h, SD.h, OneWire.h, DallasTemperature.h and Wire.h. In order for the sensor to measure temperature wirelessly inside the plasma chamber, the arduino was powered by 9V battery. For longer exposure under plasma, the breadboard attached on Arduino tend to soften and eventually damage the electric circuit. To mitigate the degradation of Arduino setup, the device was covered in plastic, and further attached to the base plate. Figure S11b shows the effect of long exposure on the wrapped plastic. The sensor probe (DS18B20) was taped on the base plate to improve contact for efficient temperature sensing and also to shield the probe from plasma directly striking it. The IR based thermal cameras were not feasible to obtain temperature during the exposure as the glass on the lid is not IR transparent. Other techniques to measure the temperature in Plasma includes spectropyrometry and optical emmision spectrometer which is outside the scope of this work.[26,27]

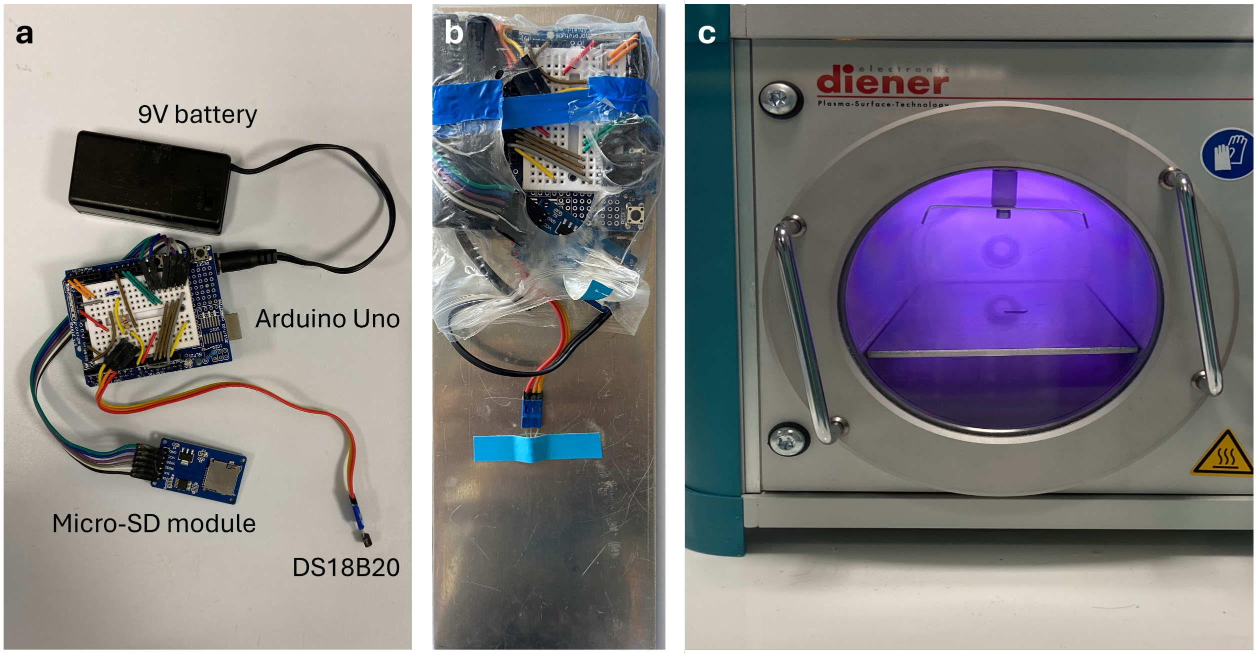

Figure S11 a) Arduino-based temperature sensor, assembled with sensor probe (DS18B20), micro-SD card reader connected to an Arduino Uno, mini-breadboard, and powered by a 9V battery. b) Thermal measurement module, wrapped in plastic to minimise damage due to direct plasma exposure, and assembled on the base plate (Al shown here). c) Al base plate resting horizontally within the cylindrical chamber of the plasma

References

[1] J. L. Fritz and M. J. Owen, *J. Adhes.*, 1995, **54**, 33–45.

[2] K. Mills, X. Zhu, S. Takayama and M. Thouless, *J. Mater. Res.*, 2008, **23**, 37–48.

[3] D. Rhee, W.-K. Lee and T. W. Odom, *Angew. Chem.*, 2017, **129**, 6623–6627.

[4] Z. Li, Y. Zhai, Y. Wang, G. M. Wendland, X. Yin and J. Xiao, *Advanced Optical Materials*, 2017, **5**, 1700425.

[5] M. J. Owen and P. J. Smith, *J Adhes Sci Technol*, 1994, **8**, 1063–1075.

[6] A. Chiche, C. M. Stafford and J. T. Cabral, *Soft Matter*, 2008, **4**, 2360–2364.

[7] F. A. Bayley, J. L. Liao, P. N. Stavrinou, A. Chiche and J. T. Cabral, *Soft Matter*, 2014, **10**, 1155–1166.

[8] M. Nania, O. K. Matar and J. T. Cabral, *Soft Matter*, 2015, **11**, 3067–3075.

[9] M. Nania, F. Foglia, O. K. Matar and J. T. Cabral, *Nanoscale*, 2017, **9**, 2030–2037.

[10] L. Pellegrino, S. Khodaparast and J. T. Cabral, *Soft Matter*, 2020, **16**, 595–603.

[11] L. Pellegrino, A. Tan and J. T. Cabral, *Physical Review Letters*, 2022, **128**, 058001.

[12] W. W. Tooley, S. Feghhi, S. J. Han, J. Wang and N. J. Sniadecki, *J. Micromech. Microeng.*, 2011, **21**, 054013.

[13] N. E. Dowling, S. L. Kampe and M. V. Kral, *Mechanical Behavior of Materials: Engineering Methods for Deformation, Fracture, and Fatigue*, Pearson, 5th edn., 2020.

[14] Y. Tsui and T. Clyne, *Thin Solid Films*, 1997, **306**, 23–33.

[15] G. G. Stoney, *Proc. R. Soc. Lond. A Math. Phys. Sci.*, 1909, **82**, 172–175.

[16] M. Hernandez, S. White, J. Chessa and C. Ramana, *Mechanics of Advanced Materials and Structures*, 2015, **22**, 1024–1030.

[17] N. S. Gupta, K.-S. Lee and A. Labouriau, *Polymers*, 2021, **13**, 1141.

[18] B. A. Grzybowski, S. T. Brittain and G. M. Whitesides, *Review of Scientific Instruments*, 1999, **70**, 2031–2037.

[19] A. F. Mills, *Heat Transfer*, CRC Press, 1992.

[20] K. Efimenko, W. E. Wallace and J. Genzer, *J. Colloid Interface Sci.*, 2002, **254**, 306 – 315.

[21] Y. Tan, B. Hu, J. Song, Z. Chu and W. Wu, *Nano-Micro Letters*, 2020, **12**, 1–42.

[22] A. Tan, L. Pellegrino and J. T. Cabral, *ACS Applied Polymer Materials*, 2021, **3**, 5162–5170.

[23] A. Tan, L. Pellegrino, Z. Ahmad and J. T. Cabral, *Adv. Opt. Mater.*, 2022, **10**, 2200964.

[24] K. Wu, T. Zhu, L. Zhu, Y. Sun, K. Chen, J. Chen, H. Yuan, Y. Wang, J. Zhang, G. Liu *et al.*, *Nano letters*, 2022, **22**, 2261–2269.

[25] A. Tan, Z. Ahmad, P. Vukusic and J. T. Cabral, *Molecules*, 2023, **28**, 1710.

[26] D. N. Meehan, K. Niemi and E. Wagenaars, *Japanese Journal of Applied Physics*, 2020, **59**, SHHB03.

[27] P.-J. Wang, C.-C. Tzeng and Y. Liu, *Advances in Optical Technologies*, 2010, **2010**, 656421.
